# RECQ5: A Mysterious Helicase at the Interface of DNA Replication and Transcription

**DOI:** 10.3390/genes11020232

**Published:** 2020-02-21

**Authors:** Martin Andrs, Zdenka Hasanova, Anna Oravetzova, Jana Dobrovolna, Pavel Janscak

**Affiliations:** 1 Institute of Molecular Genetics of the Czech Academy of Sciences, Videnska 1083, 143 00 Prague, Czech Republic; martin.andrs@img.cas.cz (M.A.); zdenka.hasanova@img.cas.cz (Z.H.); anna.oravetzova@img.cas.cz (A.O.); jana.dobrovolna@img.cas.cz (J.D.); 2 Department of Cell Biology, Charles University, Vinicna 7, 128 43 Prague, Czech Republic; 3 Institute of Molecular Cancer Research, University of Zurich, Winterthurerstrasse 190, 8057 Zurich, Switzerland

**Keywords:** RECQ5, transcription-replication conflicts, replication stress, R-loops, DNA repair, genomic instability

## Abstract

RECQ5 belongs to the RecQ family of DNA helicases. It is conserved from *Drosophila* to humans and its deficiency results in genomic instability and cancer susceptibility in mice. Human RECQ5 is known for its ability to regulate homologous recombination by disrupting RAD51 nucleoprotein filaments. It also binds to RNA polymerase II (RNAPII) and negatively regulates transcript elongation by RNAPII. Here, we summarize recent studies implicating RECQ5 in the prevention and resolution of transcription-replication conflicts, a major intrinsic source of genomic instability during cancer development.

## 1. Introduction

Helicases are a diverse group of motor proteins that participate in nearly all aspects of DNA and RNA metabolism. The RecQ helicase family belongs to helicase superfamily 2 [1], and includes enzymes that share homology with the *Escherichia coli* DNA helicase RecQ. RecQ homologues have been found in all organisms examined to date, including bacteria, yeast, plants, *Drosophila*, amphibians and mammals [2,3,4]. Mammalian genomes encode five different RecQ homologues—RECQ1, BLM (RECQ2), WRN (RECQ3), RECQ4 and RECQ5—that have essential roles in DNA replication, transcription, DNA repair and chromatin remodeling, and that are considered guardians of the genome [2,5,6,7]. Inherited mutations in the BLM, WRN and RECQ4 genes are linked to Bloom-, Werner- and Rothmund-Thomson syndrome, respectively [7]. These rare autosomal recessive disorders manifest themselves by genomic instability, premature aging, neurodegenerative and developmental defects as well as cancer predisposition [2,3,7,8].

RECQ5 is conserved from *Drosophila* to humans [9,10,11]. It has not been linked to any cancer-prone syndrome, but several lines of evidence suggest that its deficiency can cause genomic instability and cancer development [12,13,14,15]. RECQ5 has long been known to act as an anti-recombinase by disrupting RAD51 nucleoprotein filaments. Recent studies, summarized in this review article, suggest that RECQ5 plays major and unique roles in the prevention and resolution of transcription-replication conflicts (TRCs) in human cells.

## 2. Structure and Biochemical Properties of RECQ5

The alternative splicing of the *RECQ5* gene transcript yields three isoforms: α, β, and γ. The RECQ5α and RECQ5γ isoforms, comprised of an incomplete RecQ homology region (residues 1–410 and 1–435, respectively), are localized in the cytoplasm and lack helicase and ATPase activities [11,16]. The RECQ5β isoform (residues 1–991) includes a functional RecQ helicase core followed by an extended C-terminal region, which contains a nuclear localization signal. In this review, we will only discuss the nuclear RECQ5β isoform and, for simplicity, will refer to it as RECQ5.

**Figure 1:**
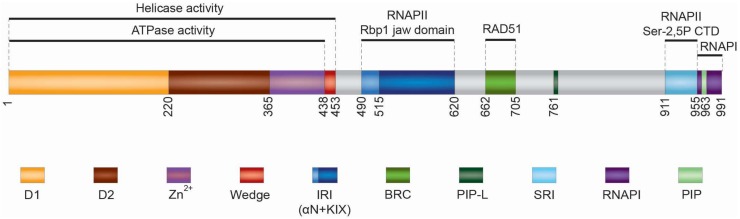
Domain organization of human RECQ5 helicase. For description of the individual domains see the main text. The positions of amino acids at domain boundaries are indicated.

RECQ5 structure can be divided into two parts: the conserved N-terminal region including the RecA-like helicase domain (residues 1–364) and Zn^2+^-binding domain (residues 365–437) [16,17], and a unique C-terminal region (residues 438–991) harboring at least five specific protein interaction domains: RAD51-binding domain, internal RNAPII-interacting (IRI) domain that binds to hypophosphorylated form of RNAPII (RNAPIIa), Set2-Rpb1-interacting (SRI) domain that binds to the hyperphosphorylated (elongating) form of RNAPII (RNAPIIo), RNA polymerase I (RNAPI)-binding domain and PCNA-interacting protein (PIP) motifs [18,19,20,21,22,23,24,25,26,27] (Figure 1).

The helicase domain consists of two RecA-like domains, D1 and D2, each containing a central 6- or 7-stranded parallel β-sheet flanked on either side by α-helices [17]. The Zn^2+^ binding domain follows immediately after and is closely associated with the D2 domain [17]. In the crystal structure of RECQ5, a single α-helix can be seen following on from the Zn^2+^ binding domain (residues 438–453), occupying a broadly similar position to the winged helix domain found in the RecQ C-terminal regions of other RecQ helicases [17]. Biochemical analysis of RECQ5 deletion variants has revealed that this structural element is essential for the helicase activity of the enzyme and has an important accessory role in DNA binding, suggesting that it may act as a wedge to aid in DNA duplex separation by the helicase [17]. Like other RecQ family members, RECQ5 functions as a dsDNA/ssDNA-dependent ATPase and an ATP-dependent 3′–5′ helicase with the ability to promote branch migration of Holliday junctions [28]. However, RECQ5 requires the single strand-binding factor RPA to mediate efficient DNA unwinding [28]. The reason for this is that RECQ5 contains a strong intrinsic DNA-strand annealing activity that resides in the C-terminal portion of the RECQ5 polypeptide [28]. This activity is inhibited by RPA and is suppressed in the ATP-bound form of the enzyme [28]. Interestingly, on DNA structures resembling a stalled replication fork, RECQ5 preferentially unwinds the lagging strand arm in a manner dependent on the region between residues 561–651 [26]. This region is not required for unwinding simple 3′-tailed DNA duplexes by RECQ5, suggesting that it may mediate RECQ5 loading on the parental duplex at fork junction in an orientation that allows enzyme translocation towards the lagging arm [26].

RECQ5 interacts with RAD51 through two conserved motifs (residues 662–705) that display a strong resemblance to those found in the BRC repeat of BRCA2, which promotes homologous recombination (HR) by mediating RAD51 filament assembly [18,19]. Interestingly, it has been shown that RECQ5 can utilize its ATP-dependent ssDNA translocase activity to dismantle RAD51-ssDNA filaments [13]. This reaction is stimulated by RPA and depends on the direct binding of RECQ5 to RAD51 [13,18].

The RECQ5 IRI domain is located in the proximal part of the C-terminal portion of the RECQ5 polypeptide (residues 490–620) [23]. Structural studies and homology modeling have revealed that it consists of a region (residues 515–620) with strong homology to the so-called KIX domain [22,24], a three-helix bundle structure with a hydrophobic core found in several transcription regulators, namely CBP, p300 or MED15 [29]. The KIX domain is preceded by an additional helix, termed αN, which is connected to the KIX motif by a loop segment [24]. Structure-function analysis has shown that the IRI domain fragment (residues 490–620) is able to bind to RNAPII, whereas neither the KIX domain nor the αN helix alone possesses this ability [24]. Moreover, it has been demonstrated that RECQ5 IRI domain binds to the Rpb1 jaw domain of RNAPII [24].

The SRI motif, a left-handed three-helix bundle, was initially discovered in the yeast histone methyltransferase Set2 and its human homolog SET2 as a binding module that specifically recognizes the Ser2-Ser5-phosphorylated C-terminal-repeat domain (CTD) of RNAPII [30,31,32], a phosphorylation state associated with the elongation stage of RNAPII transcription [33]. RECQ5 shows homology only to the SRI helices 1 and 2 (residues 910–950) that form the CTD-binding interphase [23,31]. Like Set2/SET2 SRI domain, the SRI domain of RECQ5 binds specifically to Ser2-Ser5-phosphorylated CTD peptides, and mediates RECQ5-RNAPIIo complex formation [22,23,34].

In addition to RNAPII, RECQ5 interacts with RNAPI through a domain that follows immediately downstream of the SRI domain (residues 955–991) [25]. The C-terminus of RECQ5 also contains a conserved PIP motif (residues 964–971). Interestingly, between the BRC and SRI domains, RECQ5 contains another PIP-like (PIP-L) motif (residues 761–768), which lacks the conserved glutamate (Q) of PIP and, in contrast to PIP, is essential for stable binding of RECQ5 to PCNA [26,27].

Last but not least, RECQ5 was shown to physically interact with the structure-specific endonuclease MUS81-EME1 through contacts with the MUS81 subunit, and to stimulate 3′-flap DNA cleavage by MUS81-EME1 in the absence of ATP [35]. Interestingly, the stable binding of RECQ5 to MUS81-EME1 depends on its helicase domain and on the proximal part of the KIX domain (residues 515–570 including Helix-1) of RECQ5 [22,24,35]. Deletion of the latter region impairs, in addition to MUS81 binding, the ability of RECQ5 to stimulate 3′-flap DNA cleavage by MUS81-EME1, suggesting that RECQ5 stimulates the catalytic activity of the MUS81 endonuclease through direct physical interaction [35].

## 3. RECQ5 Regulates Homologous Recombination

Homologous recombination (HR) is an evolutionarily conserved process used by cells to accurately repair DNA double-strand breaks (DSBs) [36,37,38]. Briefly, HR is initiated by DNA end resection, which generates 3′-ssDNA from broken DNA ends for the RAD51 recombinase to form a nucleoprotein filament. The RAD51 filament conducts the search for homologous DNA sequence and catalyzes the invasion of the donor sister chromatid at the site of homology, resulting in the formation of a joined DNA molecule termed displacement loop (D-loop). After DNA synthesis primed by the invading DNA strand, repair can proceed via two main sub-pathways referred to as the canonical DSB repair (DSBR) and synthesis-dependent strand annealing (SDSA). In the DSBR pathway, the second DSB end is captured by the D-loop to form an intermediate with two Holliday junctions, referred to as a double Holliday junction (dHJ). This joint DNA molecule can be either resolved by specialized structure-specific endonucleases (e.g., GEN1, MUS81/EME1) into crossover (CO) or non-crossover (NCO) products, or dissolved by concerted actions of the BLM helicase and the DNA topoisomerase IIIα (TOPOIIIα), giving rise exclusively to NCO products. In the SDSA pathway, the extended D-loop is disrupted by a DNA helicase, such as RTEL1, and the newly-synthesized DNA is annealed by RAD52 to the ssDNA tail of the other break end, which is followed by gap-filling DNA synthesis and ligation. Evidence suggests that this sub-pathway of HR is favored in mitotic cells to avoid crossing over, which may give rise to chromosomal rearrangements [39].

**Figure 2:**
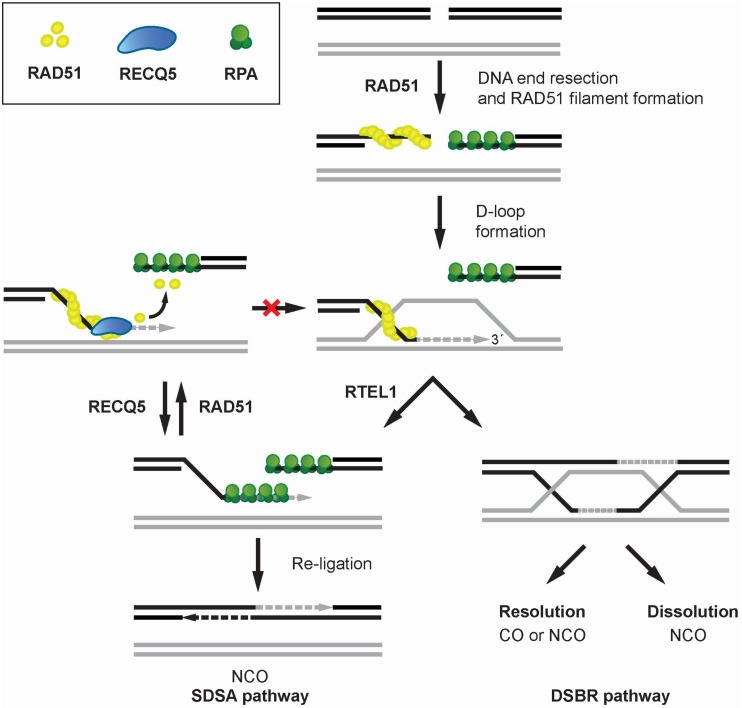
Proposed model for the role of RECQ5 in the suppression of crossovers during homologous recombination repair. RECQ5 promotes the SDSA sub-pathway of HR by disrupting RAD51 filaments formed on ssDNA after RTEL1-mediated unwinding of the extended D-loop. This prevents D-loop reassembly and its conversion to double-Holliday junction, which can be resolved to crossover (CO) or non-crossover (NCO) products.

The findings that RECQ5 possess the ability to disrupt the interaction of RAD51 with ssDNA and to suppress HR-mediated DSB-repair with CO outcomes led initially to the proposal that RECQ5 inhibits the initiation of HR by disrupting RAD51 presynaptic filaments [12,13]. This model is consistent with the observation of rapid recruitment of RECQ5 to sites of laser-induced DSBs occurring in a manner dependent on the presence of the MRE11-RAD50-NBS1 (MRN) complex that mediates DNA-end resection [40]. Furthermore, it was shown that overexpression of RECQ5 inhibits DSB repair by HR with NCO outcomes [18]. However, a more recent study revealed that HR with NCO outcomes is also impaired in RECQ5-depleted cells [41]. Moreover, the authors found that RECQ5 depletion increases sister chromatid exchange frequency upon the inactivation of the dHJ dissolution pathway by BLM depletion or upon induction of extensive DNA damage [41]. Similar results were obtained earlier with chicken DT40 cells [42]. The former study also revealed that RECQ5 deficiency increases RAD51 occupancy at DSB sites [41]. Taken together, these findings suggest that RECQ5 acts during the post-synaptic phase of SDSA to prevent formation of aberrant RAD51 filaments on the extended invading strand, thus limiting its channeling into potentially hazardous crossover pathway of HR (Figure 2). In agreement with this model, RECQ5 can utilize its ATPase and RAD51-binding activities to counteract the inhibitory effects of RAD51 and BRCA2 on DSB repair by single-strand annealing that resembles the post-synaptic steps of SDSA [41]. Moreover, RECQ5 can also alleviate the inhibitory effect of RAD51 on RAD52-mediated ssDNA annealing in vitro [41].

## 4. RECQ5 Regulates Transcription Elongation and Prevents Transcription Stress

Chromatin immunoprecipitation experiments have shown that RECQ5 associates with the coding regions of RNAPII-transcribed genes in a manner dependent on its SRI motif, with RECQ5 density correlating with the density of Ser2-CTD phosphorylation, suggesting that RECQ5 binds to the RNAPII complex during the productive elongation phase of transcription [23,43]. Loss of this interaction is associated with transcription-dependent genomic instability [34]. Interestingly, it was also reported that the transcription of the genes associated with RECQ5 increased upon RECQ5 depletion, suggesting that RECQ5 regulates RNAPII transcription [43]. Indeed, experiments with a reconstituted transcription system containing RNAPII and basal transcription factors revealed that RECQ5 inhibits transcript elongation by RNAPII in a manner dependent on its binding to RNAPII via the IRI domain [21]. This inhibitory effect on RNAPII transcription does not require the ATPase/helicase activity of RECQ5, but depends on the presence of its helicase core domain, even though only the IRI domain physically interacts with RNAPII [21,24]. Further insight into the molecular mechanism of RNAPII transcription repression by RECQ5 has been provided by a structural analysis of the complex between an elongating RNAPII and RECQ5 using cryo-electron microscopy (cryo-EM) [24]. 3D reconstruction of the complex has revealed that the IRI domain of RECQ5 is bound to the Rpb1 jaw domain in such a way that the helicase domain is located in a position that allows it to engage with DNA entering the RNAPII catalytic cleft, presenting a steric block to elongation [24]. Interestingly, structural studies conducted by Kassube et al. also revealed that the KIX domain of RECQ5 resembles domain II of the transcription elongation factor TFIIS, which binds to the Rpb1 jaw domain of RNAPII near the RECQ5-binding site [24]. Biochemical analysis demonstrated that RECQ5 IRI domain and TFIIS compete for binding to RNAPII [24]. Moreover, RECQ5 was found to interfere with the ability of TFIIS to promote transcription elongation on a DNA template containing intrinsic transcription stall sites [24]. This activity of RECQ5 requires binding of its IRI domain to RNAPII but it is independent of the helicase domain of RECQ5. Collectively, these findings suggest that RECQ5 regulates transcript elongation by two different activities resulting from the binding of its IRI domain to the Rpb1 jaw domain of the elongating transcription complex: (i) steric blockage of elongation by interaction between RECQ5 helicase domain and the DNA template; (ii) inhibition of TFIIS function. Kassube et al. proposed that by these coordinated activities, RECQ5 could prevent transcription at sites of DNA replication and DNA repair [24]. Although the SRI domain of RECQ5 is not essential for RECQ5-mediated inhibition of RNAPII transcription in vitro, the authors suggested that this domain might prime RECQ5 for engaging specifically with elongating RNAPII [24]. Of note, mutational inactivation of the SRI motif of RECQ5 abolishes its interaction with RNAPIIo but not RNAPIIa, while disruption of the IRI domain abolishes RECQ5 binding to RNAPIIa, but not to RNAPIIo [22,23]. This suggests that RNAPII CTD phosphorylation generates a form of RNAPII with low binding affinity for the IRI domain of RECQ5. Thus, it is possible that the SRI domain of RECQ5 may facilitate the binding of its IRI domain to elongating RNAPII by providing an additional tethering site.

A more recent study provided evidence that RECQ5 acts as a general transcription factor that is important for the maintenance of genomic stability during transcription in human cells [44]. This study demonstrated that RECQ5 depletion causes a marked increase in transcript elongation rates genome-wide, which is accompanied by increased frequency of incidents during which the polymerase is paused or arrested in the transcribed region of genes, a phenomenon termed transcription stress [44]. Moreover, it was shown that loss of RECQ5 results in genomic instability associated with the transcribed regions of genes and common fragile sites (CFSs), with chromosomal breakpoints overlapping with the areas of elevated transcription stress [44]. Based on these findings, the authors proposed that RECQ5 regulates transcript elongation by RNAPII to prevent transcription stress and subsequent genomic instability. However, it remains to be determined whether RECQ5 suppresses transcription stress through the direct binding to RNAPII via its IRI and SRI domains. Another question that arises is how does transcription stress caused by RECQ5 deficiency give rise to genomic instability? Saponaro et al. proposed that it is not transcription stress in itself, but rather conflicts of impaired transcription complexes with the DNA replication machinery that cause genomic instability in RECQ5-deficient cells [44]. This notion is supported by a more recent observation that RECQ5 depletion causes replisome stalling in actively transcribed genes [25]. Moreover, evidence suggests that conflicts between transcription and replication machineries are the cause of genomic instability at CFSs [45]. In addition, RECQ5 was shown to associate with the transcription machinery at DNA replication foci [25].

As mentioned above, RECQ5 also stably binds to RNAPI through a domain located at its C-terminus adjacent to the SRI motif [25]. The study demonstrated that RECQ5 associates with RNAPI during transcription elongation and its depletion increases the density of RNAPI in the pre-ribosomal(r)RNA coding region, suggesting that RECQ5 might counteract RNAPI transcription stalling [25]. As in the case of RNAPII transcription, RECQ5 depletion causes replisome stalling and amplification of DNA segments in the pre-rRNA coding regions [25]. Thus, it appears that RECQ5 might have similar functions in both RNAPI and RNAPII transcription. However, it should be noted that some of the reported phenotypes of RECQ5 deficiency are also consistent with a role of RECQ5 in TRC resolution, which is discussed below.

## 5. RECQ5 Promotes Resolution of Transcription-Replication Conflicts

Accumulating evidence suggests that TRCs represent a major source of replication stress and genomic instability during cancer development [46,47]. Replication fork stalling induced by transcription results from the formation of a RNA:DNA hybrid structure, called R-loop, which is generated co-transcriptionally by invasion of the nascent transcript into the DNA duplex behind the transcription complex [48,49]. TRC-associated R-loops have been shown to be responsible for the instability of a subset of CFSs including FRA3B, FRA16D or FRA7K [45]. Interestingly, a recent study has shown that RECQ5 is essential for mitotic DNA synthesis (MiDAS) [35], a phenomenon occurring in mitotic prophase to prevent chromosome missegregation caused by under-replicated DNA in difficult-to replicate loci such as CFSs [50]. MiDAS requires proteins known to mediate the restart of stalled replication forks, namely MUS81-EME1 endonuclease, the SLX4 scaffold protein, RAD52 single-strand annealing protein and the non-catalytic subunit of DNA polymerase δ, POLD3 [50,51], and is suppressed by RAD51 and BRCA2 that, in addition to their role in HR, are known to protect stalled forks against nucleolytic processing [51,52]. The study by Di Marco et al. provided several lines of evidence suggesting that RECQ5 disrupts RAD51 filaments on stalled replication forks at CFSs to facilitate fork cleavage by MUS81/EME1 endonuclease, which triggers MiDAS [35]. RECQ5 was found to be recruited to CFSs specifically during early mitosis in a manner dependent on its phosphorylation by CDK1 and direct binding to MUS81 [35]. Moreover, RECQ5 depletion causes CFS-associated chromosome segregation defects and genomic instability [35]. Of note, a recent study conducted by Chappidi et al. demonstrated that MiDAS depends on the formation of R-loops [53]. Thus, it is possible that the genomic rearrangements at CFSs observed by Saponaro et al. in RECQ5-depleted cells might at least in part arise from a failure to restart R-loop-stalled replication forks during early mitosis.

Chappidi et al. also analyzed the molecular mechanisms underlying the processing of R-loop-stalled forks during S-phase in cells exposed to drugs that promote R-loop formation and hence enhance the frequency of TRCs in the cell [53]. This study demonstrated that R-loops induce RAD51-dependent fork reversal [53], a DNA transaction that mediates the stabilization of stalled replication forks [54,55]. Moreover, it was shown that R-loop-induced fork stalling is followed by the restart of semiconservative DNA replication mediated by RECQ5 and other proteins involved in MiDAS [53]. Further analysis of the underlying molecular mechanism revealed that RECQ5 eliminates RAD51 filaments assembled on stalled forks after RECQ1-mediated reverse branch migration, preventing another round of fork reversal and facilitating fork cleavage by MUS81-EME1 in complex with SLX4 [53]. This suggests that RECQ5 is part of the mechanism mediating the switch between replication fork stalling and restart upon TRCs. Interestingly, Chappidi et al. also identified the DNA ligase 4(LIG4)/XRCC4 complex as a new factor essential for the restart of R-loop-stalled forks [53]. In this study, it was demonstrated that replication restart requires the catalytic activity of LIG4/XRCC4, and that LIG4 deficiency leads to accumulation of MUS81-dependent DNA breaks after induction of R-loop formation [53]. Moreover, the resumption of DNA synthesis after R-loop-induced fork stalling was found to be dependent on active transcription and the presence of the transcription elongation factor ELL implicated in reactivation of arrested transcription complexes [53,56]. ELL as well as MUS81, RAD52 and LIG4 were also required for resumption of efficient transcription following induction of R-loops [53]. These findings suggested a model where fork cleavage by MUS81-EME1 relieves the torsional stress generated by TRC and hence facilitates ELL-dependent transcription restart. After fork religation mediated by RAD52 and LIG4/XRCC4, the reactivated transcription complex can pass through the replication-stalling site, allowing replication restart. Thus, this fork cleavage-religation cycle, which resembles the action of a DNA topoisomerase, would allow sequential restart of transcription and replication stalled by topological constraints generated by head-on TRC. In this way, TRCs could be resolved without disruption of transcription complexes.

**Figure 3:**
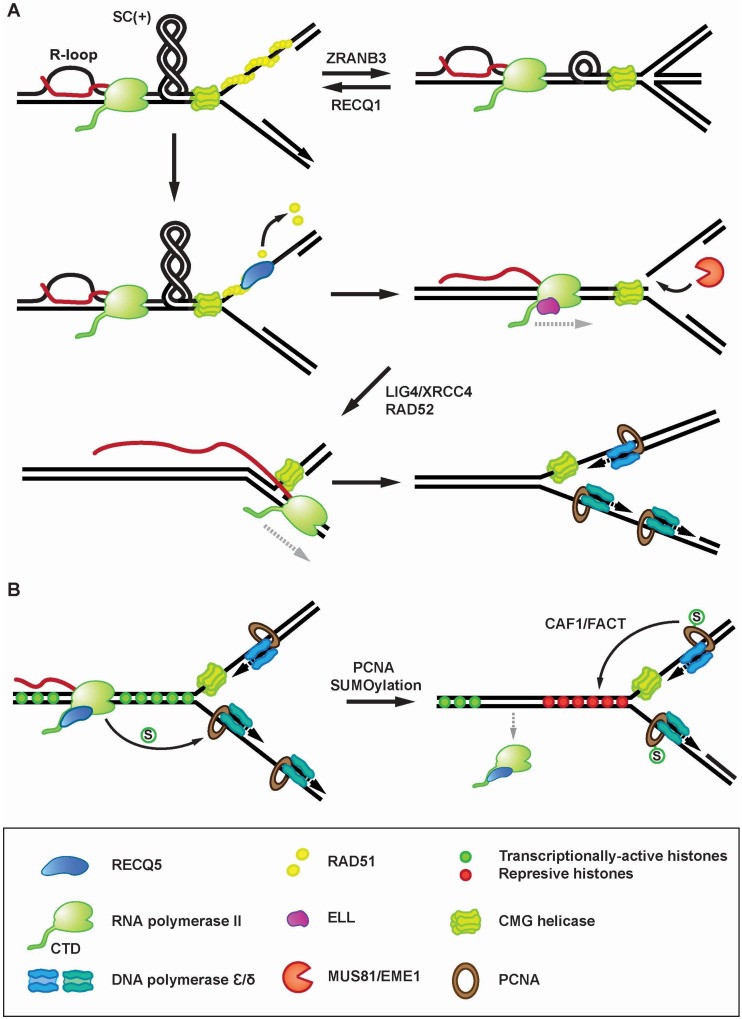
Proposed roles for RECQ5 in resolution of transcription-replication conflicts. (**A**) Role of RECQ5 in resolution of R-loop-mediated TRCs. Head-on TRCs can promote R-loop formation followed by the build-up of positively-supercoiled (+SC) domains between converging transcription- and replication machineries, which will cause replication fork stalling. Stalled replication forks are protected by the RAD51 filament, which can promote fork reversal by DNA translocase ZRANB3. Fork reversal is counteracted by RECQ1 helicase. RECQ5 helicase disrupts the RAD51 filament to prevent fork reversal and to facilitate fork cleavage by MUS81 endonuclease, which releases the topological barrier in the DNA template and triggers reactivation of transcription by ELL. After fork religation by RAD52 and LIG4/XRCC4, the reactivated transcription complex can bypass the replication-stalling site and continue RNA synthesis on the lagging arm of the fork. This is followed by replisome reloading and restart of DNA synthesis. It is assumed that after fork stalling, the replicative helicase CMG traverses the fork junction onto dsDNA via its ssDNA gate [59]. After fork religation, CMG translocates back onto ssDNA to allow the passage of the transcription complex and to nucleate a functional replisome. (**B**) TRC resolution mediated by RECQ5-dependent PCNA SUMOylation. Interaction of RNAPIIo-bound RECQ5 with PCNA triggers the PCNA SUMO2 conjugation. SUMO2-PCNA enriches histone chaperones CAF1 and FACT at replication forks to deposit repressive histones, causing RNAPII dissociation.

Another mechanism proposed for TRCs resolution involves the temporal removal of RNAPII without dissociation of RNA transcript, allowing replication fork passage [57]. Interestingly, a recent study by Li et al. revealed that RECQ5 mediates transcription-dependent conjugation of SUMO2 to PCNA, which increases the rate of replication fork progression and reduces RNAPIIo chromatin occupancy [27]. SUMO2-PCNA formation requires direct binding of RECQ5 to PCNA through the PIP-L motif [27]. Moreover, it was demonstrated that SUMO2-PCNA enriches the histone chaperons CAF1 and FACT in the replisome complex via its interaction with their SUMO-interacting motifs, enhances CAF1-dependent deposition of repressive histones at CFSs and reduces open chromatin structure, a state that likely causes destabilization of RNAPII transcription complexes [27]. These findings led to the proposal that RECQ5 in association with RNAPII acts as a sensor for replication fork to detect the oncoming transcription machinery and to trigger chromatin-remodeling pathway, leading to RNAPII eviction [27] (Figure 3B). Of note, RECQ5 was also shown to promote SUMOylation of DNA topoisomerase I that is required for its binding to RNAPIIo and for the recruitment of splicing factors to actively transcribed chromatin to prevent formation of R-loops [58]. Thus, RECQ5 is emerging as a multifunctional factor with roles in both prevention and resolution of TRCs.

## 6. Concluding Remarks

Growing evidence suggests that, in addition to its regulatory role in HR-mediated DSB repair, RECQ5 directly regulates transcript elongation by RNAPII and suppresses genomic instability associated with transcription stress. Furthermore, RECQ5 promotes TRC resolution by two different mechanisms: (i) disrupting RAD51 filaments on R-loop-stalled forks to prevent fork reversal and to facilitate fork cleavage by MUS81 endonuclease, which triggers a sequential restart of transcription and replication; and (ii) promoting PCNA SUMO2 conjugation that mediates chromatin remodeling by CAF1 and FACT leading to RNAPII dissociation. Thus, it is possible that the elevated transcription stress observed in RECQ5-depleted cells might results from defective resolution of TRCs rather than from loss of transcript elongation control by RECQ5. To answer this question, it will be necessary to analyze transcription elongation profiles in cell lines expressing relevant mutants of RECQ5. If RECQ5 suppresses transcription stress by regulating transcript elongation, one would expect to observe this stress also in cells expressing RECQ5 KIX mutants. On the other hand, if transcription stress in RECQ5-deficient cells is a consequence of unresolved TRCs, it should also be seen in cells expressing RECQ5 mutants that cannot disrupt RAD51 filaments such as RECQ5-K58R (ATPase mutant) or RECQ5-F666A (RAD51-binding mutant) [35].

It would also be interesting to learn why cells need two different mechanisms of TRC resolution, both of which require RECQ5? Which pathway is preferred? It has been shown that the RECQ5-MUS81 axis mediates resolution of TRCs induced by the DNA topoisomerase I inhibitor camptothecin (CPT), which promotes R-loop formation [53]. Interestingly, although CPT induces R-loop-mediated fork stalling [53], it does not increase PCNA-SUMO2 levels in chromatin [27]. Thus, one can speculate the RECQ5-PCNA-SUMO2 axis might promote resolution of TRCs that are not associated with R-loop formation. In such a scenario, frontal collisions would be expected, where RNAPII-bound RECQ5 would be placed in close proximity to PCNA in the replisome. Encounters between replisome and an R-loop-forming transcription complex may not lead to a frontal collision because of the buildup of topological barriers in the DNA template, preventing the interaction of RNAPII-bound RECQ5 with PCNA, which is required for PCNA SUMOylation. Much additional work will be required to fully understand the molecular processes taking place during transcription-replication interference.

## Abbreviations

BRC: internal repeats in the BRCA2 protein sequence; CFSs, common fragile sites; CMG, Cdc45-MCM-GINS; CO, crossover; CPT, camptothecin; CTD, C-terminal-repeat domain of RNAPII; D-loop, displacement loop; dHJ, double Holliday junction; DSBs, double-strand breaks; DSBR, DSB repair; dsDNA, double-stranded DNA; HR, homologous recombination; IRI, internal RNAPII-interacting domain; KIX, kinase-inducible domain interacting domain; MiDAS, mitotic DNA synthesis; MRN, MRE11-RAD50-NBS1; NCO, non-crossover; PCNA, proliferating cell nuclear antigen; PIP, PCNA-interacting protein; PIP-L, PIP-like motif; RNAPI, RNA polymerase I; RNAPII, RNA polymerase II; RNAPIIa, hypophosphorylated form of RNAPII; RNAPIIo, hyperphosphorylated form of RNAPII; SDSA, synthesis-dependent strand annealing; SRI, Set2-Rbp1-interacting domain; ssDNA, single-stranded DNA; TRC, transcription-replication conflict.
